# Advancing Research, Education, and Clinical Practice: Insights from Evidence-Based Care and Interdisciplinary Scholarship.

**DOI:** 10.51894/001c.144376

**Published:** 2025-08-29

**Authors:** Francis O. Akenami, Rana Ismail, Andrea Amalfitano

**Affiliations:** 1 College of Osteopathic Medicine – Graduate Medical Education Alliance Michigan State University, East Lansing, MI 48824.; 2 College of Osteopathic Medicine – Graduate Medical Education Alliance Michigan State University, East Lansing, MI 48824

**Keywords:** Emergency Medicine, Maternal-Fetal Health, Gastroenterology, Orthopedics, Surgical Innovation, Clinical Audit, Medical Education

## INTRODUCTION

The *Spartan Medical Research Journal (SMRJ)* presents a new collection of studies that exemplify our mission to highlight impactful, interdisciplinary scholarship. This issue features diverse contributions across emergency medicine, gastroenterology, obstetrics, orthopedics, biomaterials, and quality improvement. Each article underscores the journal’s commitment to advancing evidence-based care, educational innovation, and patient-centered outcomes.^1–6^

## Enhancing Clinical Skills in Emergency Medicine

Durell and Hooley examined the *Utility of Combining a Simulation-Based Method with Lecture for Retinopathy Training in Emergency Medicine Residency*.^1^ Their prospective pilot study demonstrated that combining lecture-based instruction with simulation significantly improved residents’ diagnostic accuracy in identifying funduscopic pathologies. Importantly, seniority and prior ophthalmology exposure did not influence outcomes, suggesting that structured simulation can serve as a universal equalizer in medical training.

## Maternal-Fetal Health and COVID-19

Rajendiran et al. investigated the *Association between Timing of COVID-19 Diagnosis in Pregnancy and Maternal-Fetal Outcomes*.^2^ Their retrospective study of nearly 2,000 pregnancies found that third-trimester infection was associated with the highest rates of placental abnormalities and preterm delivery. These findings emphasize the necessity of heightened monitoring protocols and tailored perinatal care during pandemics and public health emergencies.

## Gastroenterology and Risk Stratification

Neamah, Davies, and colleagues explored the *Glasgow Blatchford Score (GBS)* in patients with upper gastrointestinal bleeding.^3^ Their results demonstrated that a GBS greater than 10 was strongly correlated with the need for transfusion and hemostatic intervention. However, the authors also caution against rigid application of thresholds, advocating instead for nuanced use of GBS in diverse clinical contexts.

## Orthopedic Advances in Patella Fracture Fixation

Archutowski et al. presented a *Retrospective Case Series on Suture Button with Tension Band Fixation for Patella Fractures*.^4^ The study reported high rates of fracture union and reduced need for hardware removal compared with traditional wire-based constructs. Their findings provide orthopedic surgeons with a promising alternative for cases complicated by fracture comminution or symptomatic hardware.

## Innovations in Biomaterials

Expanding on musculoskeletal innovation, Daungsupawong and Wiwanitkit contributed a *Correspondence on the Influence of Porosities of 3D-Printed Titanium Implants*.^5^ Their commentary underscores the importance of larger sample sizes, longer follow-up, and inclusion of multiple control groups to enhance reliability. They also propose exploring hybrid materials and coatings to optimize tendon integration and regenerative outcomes.

## Quality Improvement in Urology

Closing this issue, Enemoh et al. reported on a *Two-Cycle Audit of Serum Calcium and Urate Testing in Patients with Renal Stones*.^6^ Through a combination of educational sessions, reminder systems, and structured feedback, the team achieved 100% compliance in calcium testing and a sixfold increase in urate testing. Their success story illustrates how targeted quality improvement interventions can bring measurable change in adherence to clinical guidelines.

## CONCLUSION

Together, these contributions reflect the breadth and rigor of contemporary medical research. From simulation-based training and risk prediction models to orthopedic innovation and audit-driven improvement, each study reinforces the journal’s mission: to bridge science with practice and to foster advances that improve patient care. We extend gratitude to our authors, reviewers, and readers for their continued support of SMRJ as a platform for scholarly excellence.
